# Morphological and chemical changes in nuclear graphite target under vacuum and high-temperature conditions

**DOI:** 10.1016/j.heliyon.2024.e32718

**Published:** 2024-06-08

**Authors:** Stefania De Rosa, Elisabetta Colantoni, Paolo Branchini, Domizia Orestano, Antonio Passeri, Gianlorenzo Bussetti, Lisa Centofante, Stefano Corradetti, Martina Marsotto, Chiara Battocchio, Cristina Riccucci, Luca Tortora

**Affiliations:** a LASR3 Surface Analysis Laboratory Roma Tre, via della Vasca Navale 84, Rome, Italy; b INFN, Roma Tre, via della Vasca Navale 84, Rome, Italy; c Department of Mathematics and Physics, Roma Tre University, via della Vasca Navale 84, Rome, Italy; d Department of Physics, Politecnico di Milano, Piazza Leonardo da Vinci 32, I-20133 Milano, Italy; e Legnaro National Laboratories (INFN-LNL), Viale dell’Università 2, Legnaro, Padova, Italy; f Department of Sciences, Roma Tre University, Via della Vasca Navale 84, Rome, Italy; g CNR- ISMN, Institute for the Study of Nanostructured Materials, Via Salaria Km 29300, Montelibretti, Rome, Italy

**Keywords:** Nuclear graphite, High-energy reactions, EDM, HTR, MSR

## Abstract

Nuclear-grade graphite is a high-efficiency material, widely used for vacuum applications in nuclear reactors and accelerators as targets facing particle beams. In these contexts, graphite is often exposed to extreme thermal stresses altering its physical and chemical properties. The thermal-induced release of volatile contaminants from targets and the damage of structural components are critical issues that can affect the safety and operation efficiency of beamline facilities. Here, we provide for the first time a detailed picture of the chemical and morphological changes occurring in a nuclear-grade graphite target, obtained through Electrical Discharge Machining (EDM), when exposed in vacuum to high temperatures. The radial temperature gradient induced by the impact of a pulsed energetic (MeV- GeV range) focused particle beams was reproduced by cyclically heating, in the 1300–1800 K temperature range, a disc-shaped graphite target in a vacuum setup. An accurate surface and in-depth chemical analysis of the graphite target was obtained thanks to the high sensitivity (ppm/ppb) of the Time-of-Flight Secondary Ion Mass Spectrometry (ToF-SIMS) technique. The chemical maps clearly show the presence of several metal oxides and impurities in the surface and subsurface regions of the untreated sample. Such contaminants were removed because of the thermal treatment in vacuum more or less efficiently, as demonstrated by Thermogravimetric analysis (TGA), X-ray Photoelectron Spectroscopy (XPS), and ToF-SIMS. However, Raman spectroscopy and SEM-EDS revealed that the high-temperature treatment induces a decrease in the crystallite size of the graphite as well as changes in the target surface porosity with the appearance of microvoids, leading the graphite target to be more prone to the breakage.

## 1 Introduction

Graphite is a widespread choice for several scientific and industrial applications in virtue of its nominal properties, such as high resistance to heat, chemical stability, mechanical hardness, and good electrical and thermal conductivity. Synthetic graphite is used for different technological purposes including electronic devices, batteries, and aerospace components [[1], [2], [3], [4], [5], [6]]. High-purity nuclear-grade graphite plays an important role in nuclear reactors, in which it is employed either as a particle moderator or as a structural material [[7], [8], [9]]. Moreover, due to the low atomic number of graphite, its well-known capability to survive high neutron fluences [10,11], and good pion production efficiency, in various particle accelerators nuclear-grade graphite is a constituent material for beamline targets [[12], [13], [14], [15], [16]]. In these facilities, targets are irradiated with pulsed high-energy particle beams and thus are exposed to extreme thermal stresses and localized temperature gradients. When the microstructural properties of graphite are altered by irradiation, the target performance can be compromised [13,[17], [18], [19], [20]], leading to macroscopic phenomena like material swelling and mass loss at high temperatures, responsible for severe breaking [19,[21], [22], [23]]. These effects were extensively studied in works where nuclear-grade graphite is used for structural components in nuclear reactors [[24], [25], [26], [27]]. The graphite damage evolution was often examined as a function of the increasing temperature [[28], [29], [30], [31], [32]], and several thermal treatments were used to clean contaminated samples [33,34]. The compositional properties of graphite play a role in its thermal behavior (e.g., emissivity, thermal conductivity) and mechanical properties (e.g., stress limit), and the evaluation of contaminants in graphite target and ancillary components, such as beam windows or collimators, is of extreme importance to assess the potential contamination of the beamline and the correlated radioprotection, and safety issues [35]. All the target manufacturing processes, including cutting and finishing procedures, are sources of contamination that must be carefully considered. Electrical Discharge Machining (EDM) stands as a pivotal technique in the precision engineering landscape, offering unparalleled capabilities in shaping complex materials, such as graphite, ceramics, and hard metals. The realization of graphite targets through EDM, while instrumental in achieving intricate geometries and tight tolerances, introduces a multifaceted challenge related to induced contamination on the graphite surfaces [36,37]. This concern arises from the intricate series of electrical discharges that occur during the machining process, leading to unintended alterations in the surface properties of the graphite material. As EDM progresses, the interaction between the tool and workpiece in the presence of dielectric fluid triggers the thermal decomposition of dielectrics, releasing toxic vapors, fumes, and aerosols [38]. This phenomenon, often overlooked, poses a critical threat to the pristine nature of graphite surfaces and compromises the integrity of the fabricated sample. The contamination-induced alterations in the surface characteristics of graphite can have far-reaching implications on its thermal, electrical, and mechanical properties, ultimately impacting the performance and reliability of the material.

In particular, during the wire EDM procedure, the surface of the wire can volatilize, leaving a recast layer on the surface of the work material [39]. Different foreign elements are expected to diffuse into the workpiece, in addition to the deposition of oxygen on the cut surface [40]. For example, a recast layer consisting of Cu and Zn residues is expected when a brass wire is used for EDM [[40], [41], [42], [43]]. If the recast layer is not removed by polishing procedures, it can cause the failure of the target and safety issues in the beamlines. The starting chemical composition of the target is one of the fundamental parameters used in numerical simulations to evaluate the target response to energetic particle beams, in terms of structural integrity and emission of activated species [35,44,45]. Time-consuming mechanical and chemical polishing procedures are commonly adopted for the EDM recast layer on high-performing components [41,46]. It was recently demonstrated that graphite porosity is influenced by the change of its chemical composition and bulk oxidation [47], and a large part of the scientific literature is devoted to studying how different oxidation routines in air can induce changes in the graphite microstructure (e.g., grain size, graphitization degree) [31,[48], [49], [50]]. This work aims to fill the literature gap for nuclear-grade graphite targets subjected to cyclic thermal treatments at high temperatures in vacuum conditions or inert atmospheres. A disc-shaped target was obtained from a commercial cylinder of isostatic-molded graphite with ultra-fine grain through wire EDM technique. The target was then treated with a thermal routine reproducing the high-temperature gradient induced by the impact of pulsed high-power density particle beams. Although the heat treatment cannot faithfully simulate the effects of a pulsed highly energetic particle beam, being too slow, such approach was already successfully adopted for steady state temperature and thermal conductivity measurements, being able to create strong thermal gradients on the sample surface [22]. In addition, cyclic heat treatments were performed to investigate the potential effects of heating and cooling cycles on the sample morphology. Despite being slow, the heating and cooling cycles can be sufficiently strong to cause the warping effect on the sample. Future studies may involve the use of a more rapid heating-cooling cycle, such as the one obtained from an electron beam, to better simulate the pulsed energetic particle beam. Here, topographical, structural, and chemical properties of the material before and after the thermal treatment were investigated by combining well-established analytical techniques such as stylus profilometry (SP), Scanning Electron Microscopy (SEM-EDS), Raman spectroscopy, TGA, and XPS. Furthermore, here for the first time, ToF-SIMS experiments were adopted to study the spatial distribution of elemental and molecular impurities in graphite targets, correlating changes in chemical composition and microstructural features.

## 2 Materials and methods

### 2.1 Materials

This work was conducted on isostatic-molded graphite with ultra-fine grain (HPG-59 grade, TOYO TANSO). The disks were cut from a semifinished graphite cylinder employing a Sodick AQ-LN1W electrical discharge machine with a brass wire electrode of 250 μm. Different graphite targets with thickness ranging from 300 μm to 1 mm were obtained from a single circular graphite bar with a diameter of 40 mm. The samples analyzed in this work have a diameter of 40 mm and a thickness around 300 μm.

### 2.2 Thermal treatment

The experimental setup adopted for the thermal treatment is reported in Ref. [51], where it was used for the estimation of the thermal conductivity at high temperatures of graphite and carbide target prototypes. All the components of the apparatus are kept at approximately 10^−6^ mbar in a water-cooled vacuum chamber. A power supply (I_max_ = 1000 A, V_max_ = 10 V) heats by Joule effect a graphite heater through two water-cooled copper clamps. The heater is designed to produce on its top surface (18 mm diameter) a homogeneous temperature distribution up to 2500 K. Four tungsten bars suspend the sample disk coaxially with respect to the hot circular surface of the heater, allowing precise regulation of the spacing between the sample and the heater, ranging from 0.2 mm to 5 mm. The spacing parameter is used to tune the maximum sample temperature (in the center), and the sharpness of the temperature gradient in the radial direction. The temperature is measured with a high-temperature infrared pyrometer placed on the top of the vacuum chamber in correspondence with a boro-silicate glass window, almost completely transparent to infrared radiation. Data are collected automatically with a programmable logic controller. The graphite disk was heated five times reaching 1773 K at the center, and 1273 K at the edge at the controlled heating rate of 5 K/min. The heating phases were alternated with cooling phases down to about 1273 K at the center, and about 873 K at the edge, at the same rate as the heating phase. At the end of the cyclic treatment, the sample was brought back to room temperature. No evidence of tungsten evaporation or damage to the W bars was found during the measurements and in the previous experimental campaigns using the apparatus. The tungsten bars are not in contact with the heating crucible, and although they are directly irradiated by it, the bars reach temperatures that are way below their evaporation temperature in a high vacuum. The experimental apparatus is not designed to measure chemical or physical sample properties in situ.

## 3 Experimental

### 3.1 Stylus profilometry

Large-scale surface topography measurements were collected ex-situ with a stylus profilometer (KLA Tencor P7), equipped with a diamond tip of 0.2 μm, allowing a lateral resolution of 20 nm–50 nm. The vertical resolution and maximum range are 0.4 nm and 1 mm, respectively. 3D surface topography maps (2.5 mm × 2.5 mm) were collected with a scan speed of 200 μm/s. The applied force of the stylus was 0.50 mg. The reported RMS surface roughness values are computed by averaging at least five measurements. The collected data were further analyzed with Apex Software.

### 3.2 SEM

Scanning electron microscopy images of the morphology of the samples were taken with a Tescan Vega3 scanning electron microscope operating at 10 kV. In order to have comparable images for the three cases (untreated material, treated edge, and treated center), the same magnification and working distance (15 mm) were maintained during the analysis. The graphite samples were directly observed without any mechanical or surface treatment.

### 3.3 ToF-SIMS

ToF-SIMS analysis was performed using a ToF-SIMS V instrument (ION-TOF GmbH). The base pressure of the analysis chamber during the measurements was ∼10^−9^ mbar. Bi ^+^ primary ions at 30 keV, provided by a liquid metal ion source, were used to bombard the target surface and produce secondary ions. Mass spectra were collected in high mass resolution bunched mode, while the high lateral resolution chemical maps were obtained through the fine focusing (long pulsing) of the primary beam. The total primary ion dose reached during the mass spectra acquisition was ∼ 1 × 10^9^ ions in all experiments. Depth profiling measurements were acquired in interlaced dual-beam mode with a Cs^+^ sputter gun at 1 keV and Bi^+^ analysis beam. Secondary ions were collected from sample areas of 100 μm × 100 μm. For depth profiling experiments, the analysis area was centered in the sputtering area of 300 μm × 300 μm, to avoid edge distortion effects. Mass spectra were calibrated using H_2_^−^, C^−^, C_4_^−^, and C_8_^−^ secondary ion signals. The reported ion peak intensities were averaged on ten measurements acquired on the considered portion of the target surface. Mass spectra, chemical maps, and depth profile data were exported for further analysis by SurfaceLab v6.5 software.

### 3.4 TGA

Thermogravimetric analyses (TGA) were carried out in N_2_ inert atmosphere (gas fluxes of 100 ml/min) from room temperature to 1770 K by using an automated thermal analyzer Model SDT Q600 (TA Instruments). A 7 mg sample of HPG59 graphite was obtained by simply detaching a single fragment from the edge of the untreated sample without the use of any additional tools. No grinding or other mechanical procedures were adopted. The fragment was then placed in an alumina pan (90 μL), stabilized at room temperature, and subsequently heated with a scan rate of 5 K/min. The balance sensitivity was 0,1 μg.

### 3.5 XPS

XPS analyses were performed in a homemade instrument consisting of two chambers (preparation and analysis) separated by a gate valve. The analysis chamber is equipped with a manipulator having six degrees of freedom and with a 150-mm mean radius hemispherical electron analyzer with a five-lens output system (operating at a pass energy of 25 eV, during the experiments) combined with a 16-channel detector (Multiplier Voltage used 1950 eV). Measurements were performed at normal take-off angle (θ = 90°). Samples were introduced in the preparation chamber, left outgassing overnight at a pressure of about 10-8 mbar, and subsequently introduced in the analysis chamber. The vacuum in the analysis chamber during measurements was in the 10-9 – 10-10 mbar range. Al Kα non-monochromatized X-radiation (hω = 1486.6 eV) was used to record wide scans (1–1400 eV BE range), C1s, and O1s photoemission spectra on the samples.

### 3.6 Raman spectroscopy

For Raman spectroscopy measurements, the samples were placed under a Nikon Eclipse Ni microscope of an NT-MDT Confotec NR500 confocal Raman spectrometer. A 632.8 nm laser was exploited as an excitation source. The light was focused through a 60 × objective on the sample. No heating or surface damage to the samples was induced by the laser.

## 4 Results and discussions

**Fig. 1:**
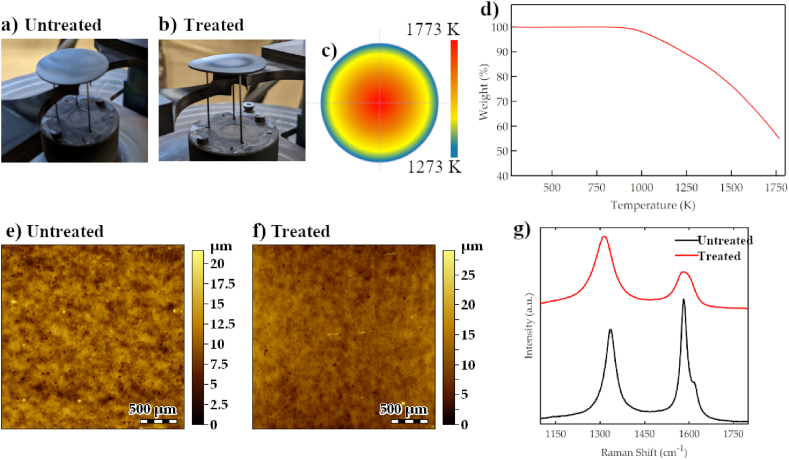
Target picture before (a), and after (b) the thermal treatment. (c) Sketch of the temperature radial gradient, with decreasing temperature values from the center to the periphery of the graphite disk. (d) Thermogravimetry weight loss curve in N_2_ inert atmosphere of untreated HPG59 graphite. Topographic surface maps from (e) untreated graphite (S_q_ = 1.60 μm), and (f) treated graphite (S_q_ = 1.68 μm). (g) Raman spectra of untreated (black curve), and treated (red curve) graphite target.

A disk of high-density percolation graphite (HPG) with a thickness of 300 μm was obtained from a semifinished graphite cylinder (∅ 40 mm) through wire cut electrical discharge machining process (Fig. 1a). The HPG graphite disk was then moved in an experimental setup for the thermal treatment where the heater is designed to produce on its top surface a homogeneous temperature distribution up to 2500 K (Fig. 1b). The sample was subjected to five cycles of heating and cooling in the temperature range 800–1800 K, generating a temperature radial gradient in the graphite disk. The strong radial temperature gradient induced in the graphitic target reproduces the temperature field due to the power deposition of a high-energy particle beam [22]. The gradient profile, schematized in Fig. 1c has a maximum value of 1773 K at the center disk, and a minimum value of 1273 K, measured at the edge of the disk. Comparing the pictures of the graphite target collected before and after the thermal treatment (Fig. 1a–and b, respectively), it is evident that a change in the sample shape, such as bending or shear deformation, occurred. As reported in the literature, such strong thermal stress may promote substantial and permanent changes in high-energy beam target materials usually due to different phenomena like mass loss, chemical reactions, or structural modifications [17,18,21,23].

### 4.1 Topographical and structural modifications

To better understand the structural deformations of the HPG graphite target observed after the treatment at high temperatures, the thermal, morphological, and structural characterization of the treated and untreated samples was performed through DTA-TGA, SEM-EDS, and Raman spectroscopy. The attention was also focused on the changes that occurred on both the center and edge of the treated graphite disk in response to the temperature radial gradient.

Potential weight losses were therefore investigated through DTA-TGA operating at the same temperature as the thermal treatment and in N_2_ inert atmosphere. The thermal stability of the untreated graphite target was tested from 298K to 1770K, using a heating ramp of 5K/min. In Fig. 1d, an intense weight loss starts at approximately 900 K, maintaining a high rate until 1770 K, where the loss value is more than 40 %. DTA signal in Fig. S1 shows the occurrence of an exothermic process around 500K followed by an endothermic process from 500K to 1250K, and finally again sample exothermic changes from 1250 to 1770K. In general, a weight loss at high temperatures around 400–500K is usually attributed to a combustion process and, in the case of graphite, to the decomposition of oxygen-containing functional groups such as –OH, C–O, C=O, and C–*O*–C. In the untreated graphite target, the oxygen groups are largely present (>30 %), as revealed by the elemental analysis (Table S1 and Fig. S2) and confirmed, later in the text, by XPS results [52,53]. However, the intense weight loss starting around 900K could be due to different effects. Firstly, at such very high temperatures, carbon atoms of graphite can be eliminated together with the oxygen during the heating process, or as reported in literature, around 1500K carbon atoms can react with nitrogen molecules leading to the formation and subsequent elimination of cyanogen and hydrogen cyanide. The strong increase of ΔT from 1400K to 1770K can be also related to an annealing process in graphite [54] as well as the decomposition of chemical compounds (mainly metal oxides) probably coming from the EDM cutting procedure. In fact, the cutting procedure can induce the volatilization of the chemical compounds constituting the brass wire electrodes and subsequent diffusion in depth of such contaminants on both sides of the 300 μm thick graphite sample. It is challenging to identify the reduction temperature of the metallic impurity species from the EDM wire, but from the literature, it occurs in the major cases in the temperature range 900–2300 K [34].

The morphological and structural changes induced in the graphitic target by the thermal treatment were observed at different scales in this work. Profilometry height maps were obtained from the central region of the untreated and treated graphite samples (Fig. 1e and f, respectively). The RMS surface roughness (S_q_) was calculated from the height maps over the entire area of 2.5 mm × 2.5 mm. It represents the root mean square of the height distribution within the evaluated area, and its value is S_q_ = 1.60 μm for the untreated target, and S_q_ = 1.68 μm for the treated sample. Despite the appearance of macroscopic shape distortion, the cyclic thermal treatment seems to have no impact on the mesoscopic morphological features of the target, as the RMS roughness values demonstrate. Nuclear-grade graphite is a polycrystalline material characterized by a certain content of pores, filler, and binder phases, depending on the original constituents and the manufacturing processes. The diversity of synthetic nuclear grades comes from the balance of the structural phases, graphitization, purity, and porosity [55,56]. These features affect the performance of graphite as the constituent material of a target and can change after additional manufacturing steps for the target production. Therefore, the structural evolution of the EDMed target was monitored before and after the thermal treatment, in that way considering the effects of the adopted cutting procedure. In Fig. 1g, Raman spectra of untreated (black curve) and treated (red curve) samples are reported. Raman measurements were performed in the central region, where the temperature reached the maximum value. Three typical features in the Raman spectra of graphitic materials are the G, D, and D′ bands, at about 1582 cm^−1^, 1350 cm^−1^, and 1620 cm^−1^, respectively [57]. In polycrystalline graphite, the D, and D’ bands are known to be characteristic of defects, growing in intensity with respect to the G band when the disorder increases. Polycrystals are composed of crystal laminae with an almost regular structure, randomly oriented in the basal plane [56]. The L_a_ parameter represents the crystal laminae size, which is inversely proportional to the intensity ratio of the D and G bands (I_D_/I_G_) [58]. Here, the spectrum of the untreated sample shows a prominent G band, representing the presence of the sp^2^ carbon network, and the two bands relating to the graphite starting content of defects after the EDM cutting procedure. The Raman spectrum acquired after the thermal treatment undergoes a visible broadening of the characteristic bands, and the crystallite size changes in the treated sample, as reported in Table 1. Such broadening of the G and D bands, connected to the change of the crystallite size L_a_ from 49 nm to 21 nm in the treated sample, strongly suggests the presence of alterations in the material structure, namely defects increase. However, it is important to highlight that Raman results provide structural information about the first microns of the sample that in this case could be characterized by the presence of residual stress [59] and strain induced by the EDM treatment. This effect could be less pronounced when moving from the surface to the deep bulk for the untreated as-machined sample.

**Table 1:** Crystallite size calculation for untreated and treated graphite.

	I_D_/I_G_	L_a_ [nm]
Untreated graphite	0.8	49
Treated graphite	1.8	21

**Fig. 2:**
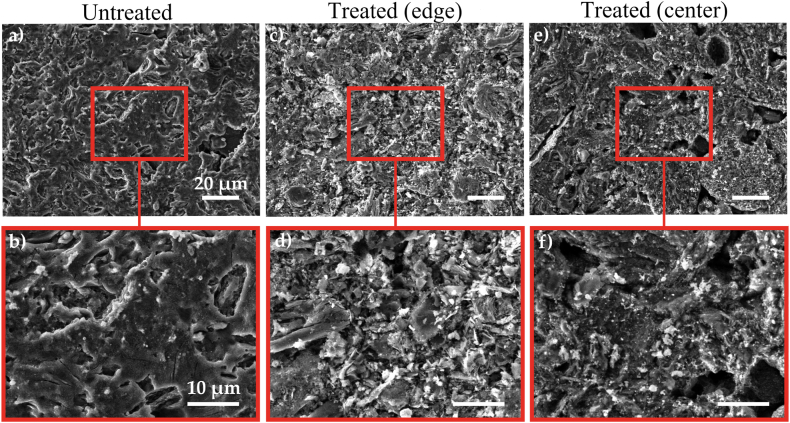
Representative SEM images at two different magnifications of (a,b) untreated graphite target, (c,d) treated graphite acquired at the target edge, and (e,f) treated graphite acquired at the target center.

These findings have practical implications for applications where surface quality and structural integrity are staples. Further confirmation regarding the presence of superficial modifications was therefore sought through the use of SEM. Some representative SEM images from the untreated and treated target surfaces are reported in Fig. 2a and b, and Fig. 2c–f, respectively, with two different magnifications. For the treated target, two representative images from the edge (∼1300 K) and the center (∼1800 K) of the disk are reported to show the morphology evolution as a function of the temperature gradient. The surface of the untreated target, in Fig. 2a and b, seems to be covered by the recast layer coming from the EDM process, therefore it does not present the well-known qualitative characteristic of nuclear-grade graphite. On the contrary, the typical features of the nuclear-grade graphite, such as micro-particles, filler/binder domains, and pores, can be easily discerned in the treated sample (see Fig. 2c–f), suggesting that the cyclic thermal treatment was able to clean the surface from contaminations. It is interesting to note the variation in pore size from a few microns in the edge of the heat-treated sample up to about tens of microns in the center of the same treated sample; this agrees with the expected effects related to the experimentally induced temperature gradient. Different SEM images were collected from the treated and untreated samples, and it is interesting to note that the target center, where the temperature reached the maximum value of 1773 K, is in general characterized by large craters and pores with sizes of tens of microns.

### 4.2 The effects of the heat treatment on the chemical impurities: surface and bulk chemical analysis

**Fig. 3:**
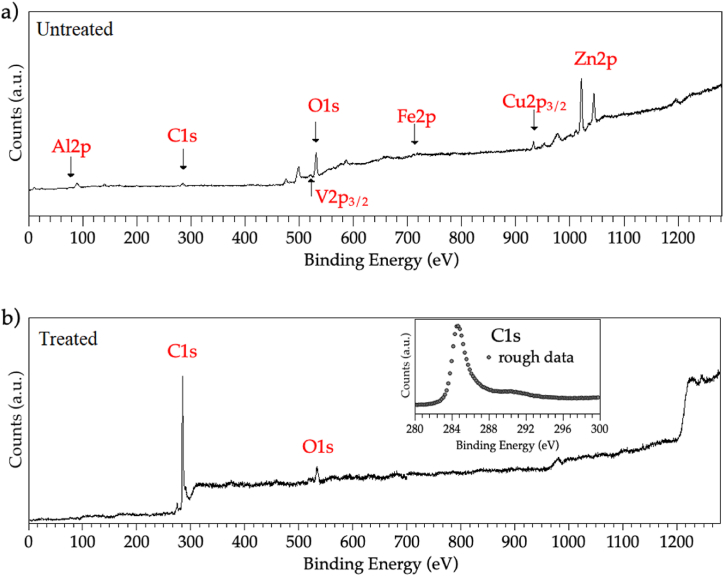
Wide scans acquired in the 1–1400 eV BE range for (a) untreated graphite, and (b) thermally treated graphite. The inset in (b) shows the C1s core-level spectrum acquired on the treated sample.

It is well-known from the literature that the pore size is one of the morphological parameters controlling the evolution of cracks in graphite exposed to mechanical or thermal stress [60]. The occurrence of strong temperature gradients may lead to the formation of areas with different porosity grades on the target, as observed in this case, promoting the target deterioration. On the other hand, the presence of the recast layer in the untreated sample must not be overlooked. When a target is bombarded with high-energy beams of particles to induce nuclear reactions and produce secondary species, the transmutation of the target material atoms into different isotope elements can also involve the impurity species. The activation of graphite impurities is an issue to take into high consideration when radioprotection procedures are planned in the beamlines. With these arguments in mind, the importance of a detailed chemical analysis of the target becomes clear. The surface chemical composition of untreated and treated graphite targets were first investigated by XPS measurements, being the XPS sampling depth of a few nanometers (less than 5 nm for most materials) [61]. The wide scans acquired in the 1–1400 eV Binding Energy (BE) range are reported in Fig. 3, showing the chemical elements individuated in the outermost layers of the surface.

The untreated sample surface (Fig. 3a) is highly contaminated by metal oxides, as indicated by the several signals labeled in the spectrum [62]. More specifically, the presence of copper (Cu) and zinc (Zn), which are the main constituents of the brass wire used in EDM, and iron (Fe) is confirmed. On the other hand, the C1s signal in the untreated sample is of very low intensity. After the thermal treatment (Fig. 3b) the spectral features related to metal elements disappear from the wide scan, and the O1s peak intensity decreases, coherently with the hypothesized purifying effect due to the thermal treatment. What is most interesting, the C1s peak in the treated sample has a very high intensity and the typical line shape expected from the literature for clean nuclear-grade graphite [33], as shown in the inset in Fig. 3b.

**Fig. 4:**
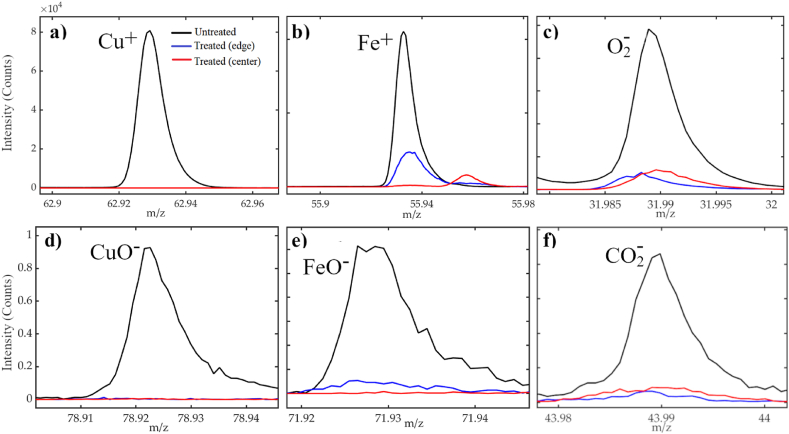
ToF-SIMS intensity peaks of copper (a), iron (b), O_2_^−^ (c), CuO^−^ (d), FeO^−^ (e), and CO_2_^−^ (f) secondary ions. The spectra were acquired on the surface of pristine graphite (black curves), the edges of heat-treated graphite (blue curves), and the center of heat-treated graphite (red curves). The primary ion dose is the same in each panel.

The presence of impurities was explored in more detail by using the ToF-SIMS technique taking advantage of its high sensitivity (ppm/ppb). It has already been proved that this technique is useful to reveal elemental and molecular surface contaminations, with low nominal concentrations, in graphitic samples [63]. Here, the chemical analysis of the target surface was performed averaging ten measurements acquired in different areas of the samples. The mass spectrum of the untreated EDMed target is characterized by the presence of metal ion peaks and oxygen-based species with high-intensity counts (∼10^4^). In Fig. 4a and b, Cu^+^ and Fe^+^ ion peaks from the untreated (black lines) and treated samples (edge-blue lines and center-red lines) are compared. As expected, the copper and iron content decreases after the thermal treatment. However, while copper completely disappears at both the center and peripheral areas of the target, iron is present in lower concentrations, on the target surface edge after the treatment. In Fig. 4c–f, some ions of metal-oxygen compounds (CuO^−^ and FeO^−^), and the O_2_^−^, CO_2_^−^ ions coming from the untreated and treated graphite surface are also reported. Surface oxidation is a crucial property that affects the efficiency and durability of a graphite-based target. Oxygen-based species can be easily generated during the machining of the graphite target, considering that EDM is a thermal process where from 2000 to 500,000 sparks per second remove material vaporizing it. Under specific conditions, carbon-oxygen chemical bonds might be formed, leading to the breaking of the graphite carbon-carbon bonds [64]. Oxidation processes can also be catalyzed by metal impurities [65]. From the ToF-SIMS surface analysis, it is possible to observe that also the oxygen-based species undergo a significant decrease in counts ascribable to the thermal treatment in vacuum (Fig. S2). For what concerns the O_2_^−^ and CO_2_^−^ ions, their intensity drastically falls in both the central and peripheral areas of the treated sample, as shown in the peak comparison of Fig. 4c–and f.

From these results, it can be assumed that, in general, after the thermal treatment, the metal and oxygen-based ion signals clearly decrease in intensity; for some of them (Cu^+^ and CuO^−^), the number of ions reaches zero counts on both the central and edge area of the graphite target while for others (Fe^+^ and FeO^−^) the decreasing seems to be inversely related to the temperature reached along the target radius. This suggests that the high temperatures reached in vacuum conditions can promote the decontamination of the target surface with different efficacy, depending on the specific elemental or molecular impurity.

**Fig. 5:**
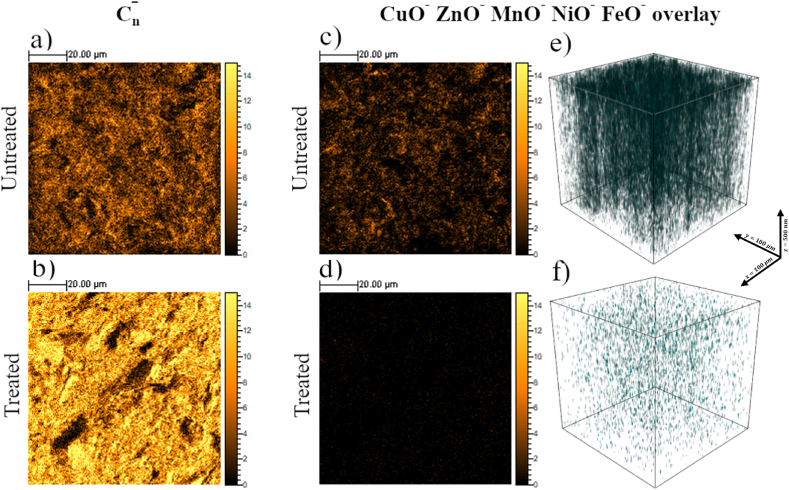
ToF-SIMS high lateral resolution ion maps (100 μm × 100 μm) showing the surface spatial distribution of the C_n_^−^ (n = 1–5) secondary ions from untreated (a), and treated (b) samples. Surface (c,d) and in-depth (e,f) spatial distribution of CuO^−^, ZnO^−^, MnO^−^, NiO^−^, and FeO^−^ secondary ion intensity overlay of untreated (c, e), and treated (d,f) graphite target.

The effect ascribed to the thermal treatment was deeply investigated with ToF-SIMS imaging analysis, by which it is possible to simultaneously obtain information about the surface morphology and the chemical composition of the analyzed surface. ToF-SIMS high lateral resolution chemical maps were not acquired due to the high roughness of the sample. The chemical maps of the carbon ion series (C_n_^−^, n = 1–5), and the signal overlay of different metal-oxide compounds from the untreated and treated sample were reported in Fig. 5a and b, and Fig. 5c and d, respectively. The chemical maps with the C_n_^−^ ion signals reproduce the graphitic surface of the target, before and after the thermal treatment. Coherently with SEM results, the C_n_^−^ ion maps are quite dissimilar in the two cases. After the thermal treatment (Fig. 5b), the chemical map of C_n_^−^ ions reveals microparticles, fractures, and pores having widths of tens of microns characteristics of the nuclear-grade graphite surface. It is interesting to note that the surface morphology of the untreated target (Fig. 5c), revealed by the secondary ion spatial distribution, is dominated by a wide range of metal-oxide compounds. The recast layer already observed with SEM is composed of Cu and Zn, as expected from the EDM brass wire, but also of other metals such as Mn, Fe, and Ni, probably present in the metal alloy in lower concentrations.

3D data-cube reconstructions in (e,f) were obtained from ToF-SIMS depth profiling experiments.

After the thermal treatment, the signal of the metal-oxide compounds homogenously decreases in intensity on the treated target surface, confirming the thermal-induced desorption of the recast layer (Fig. 5d). However, some contaminants can be still detected on the target surface, as shown in the signal spatial distribution in Fig. S4.The in-depth chemical composition of the targets was qualitatively analyzed. The presence of metal-oxide contaminations in the target bulk was followed through ToF-SIMS depth profiling experiments. This experimental configuration has been already adopted for graphitic samples [66,67]. In Fig. 5e and f, the 3D distribution of secondary ion intensities collected during the sputtering experiments is reported for the untreated and treated samples, respectively. The data cubes were obtained from the ion signals collected versus the primary ion fluence, which is proportional to the depth in samples with a homogeneous matrix. It was not possible to precisely measure the depth of the crater induced by the sputtering process due to the high surface roughness of the sample. However, a depth scale in the order of hundreds of nanometers was roughly estimated, by combining profilometry measurements and sputtering yield information previously obtained for more regular graphitic samples [66]. Notably, the signal overlay of CuO^−^, ZnO^−^, MnO^−^, NiO^−^, and FeO^−^ ion intensities from the untreated sample in Fig. 5e shows the presence of a thick recast layer that is homogeneously distributed not only on the sample surface but also in the bulk of the graphitic target; this suggests that the machining process induces not only the surface contamination of the sample but also the diffusion of the contaminants in the subsurface region for hundreds of nanometers in depth. It is well known in literature that EDM is widely adopted for the machining of stainless steel and such process induces the formation of white and heat-affected layers with a depth of hundreds of micrometers [68,69]. The white layer is mainly composed by recast and melted sublayers. In this case, the nuclear graphite, being porous in nature, is characterized by a large number of pores, craters, and cracks and it can be easily contaminated in depth by undesired subproducts of the EDM process, as also highlighted by the EDS results in Table S1 representing the concentrations of the elements near the surface. It is evident that the thermal treatment at high temperatures of the whole graphitic target causes the desorption of a large part of the metal oxides and oxygen-based species. The values of the secondary ion intensities in the 3D reconstruction of Fig. 5f do not allow us to obtain quantitative information about the impurity residual content after the thermal treatment. However, it is reasonable to relate the weight loss values from TGA measurements to the qualitative reduction of the metal oxides observed with ToF-SIMS depth profiling.

## 5 Conclusions

In this work, a disc-shaped target of commercial ultra-fine grain graphite, obtained by EDM cutting, was heated up to 1773 K with a cyclic procedure in order to reproduce the thermal stress due to the impinging of intense high-energy particle beams. The thermal stability of the as-cutted untreated graphite target was investigated by TGA analysis, which showed a significant mass loss at the adopted temperatures. This mass loss is likely due to chemical reactions that need further confirmation. It is important to note that the thermal stability is highly dependent on the environmental conditions, such as the N_2_ atmosphere used in TGA and the vacuum conditions of the cyclic heat treatments. In the untreated sample, SEM measurements revealed the presence of a surface layer hiding the expected morphological features of the nuclear graphite; such layer was supposed to be a recast layer generated during the EDM cutting procedure. In fact, the surface and, surprisingly, also the bulk chemistry of the untreated graphite sample resulted strongly modified by the EDM treatment revealing the oxidation of the graphite and the introduction of metal oxides for hundreds of nanometers in depth, as observed by XPS and ToF-SIMS analyses. When the graphitic target is then treated at high temperatures in vacuum, the recast layer seems to disappear suggesting an effective decontamination action, inherent in the heating process in vacuum; also, the pores of tens of microns, binder and filler phases, characteristic of the nuclear grade graphite, can be appreciated again. On the other hand, the thermal stress caused an increase of structural defects in the graphite sample leading to the change of the crystallite laminae size from 49 nm to 21 nm. These structural defects could also be responsible for entrapping contaminants from the EDM process, making them difficult to completely remove with heat treatment, as revealed by ToF-SIMS. As highlighted in the text, these contaminations can have significant implications for applications in high-energy particle beam environments but also nuclear reactors, and radioprotection procedures. In fact, ultra-fine grain graphite is considered a good choice also for both high-temperature reactors (HTRs) and molten salt reactors (MSRs) as molten salt–impermeable nuclear graphite compared to the fine grain graphite.

However, while our study simulates the effects of pulsed highly energetic particle beams on nuclear-grade graphite through cyclic high-temperature treatments, it is crucial to highlight the key differences between these conditions and their respective impacts on graphite structure and properties. Firstly, the thermal treatments applied in our experiments involve gradual heating and cooling cycles at a controlled rate of 5 K/min, which contrasts significantly with the rapid heating rates experienced by graphite targets under actual particle beam irradiation. For example, graphite targets are a feasible solution when designing facilities for the production of muon beams from both positron [16] and proton [12] beams. High-energy particle beams generate instantaneous localized heating, creating steep temperature gradients within microseconds. This rapid thermal cycling can lead to more severe thermal shock, inducing microcracks and other structural damages not fully replicated by slower thermal treatments. Moreover, hadron beams, especially those in the MeV-GeV range, impart kinetic energy to the graphite atoms, causing displacement damage, sputtering, and atomic dislocations. These effects result in radiation damage, where high-energy particles displace atoms from their lattice positions, leading to defects and vacancies that can significantly alter the mechanical strength, thermal conductivity, and overall stability of the graphite. Sputtering, where the impact of high-energy particles ejects surface atoms, causes erosion and changes in surface composition and roughness. Additionally, ion implantation, where incident particles become embedded in the graphite matrix, introduces foreign atoms that alter the chemical and physical properties of the material. These beam-induced damages lead to a series of changes in physical properties that are not fully captured by thermal treatments alone. Radiation-induced defects can decrease the graphite's mechanical integrity, making it more prone to fracture under stress. Displacement damage and sputtering alter the thermal conductivity and specific heat capacity of graphite, affecting its ability to dissipate heat during subsequent beam exposures. Ion implantation and defect formation can also impact the electrical conductivity of graphite, influencing its performance in applications requiring precise electrical characteristics. Linking these experimental results to practical problems, the findings from our high-temperature treatments provide valuable insights into the thermal stability and contaminant behavior of nuclear-grade graphite. However, it is important to recognize that real-world conditions in beamline facilities introduce additional complexities. Understanding the limitations of thermal treatments helps in assessing the longevity of graphite targets under actual operating conditions. While thermal treatments can pre-condition the targets and reduce initial contaminants, they cannot fully prepare the material for the extent of damage caused by particle beams. These insights highlight the importance of regular monitoring and maintenance of graphite targets to manage beam-induced damages, ensuring the safety and efficiency of beamline operations. By acknowledging and addressing these differences, our research aims to bridge the gap between laboratory simulations and practical applications, providing a more comprehensive understanding of the challenges faced by graphite targets in high-energy beam facilities.

## Fundings

The authors would like to acknowledge that funding for this work was provided by INFN within the framework RD_MUCOL. The Grant of Excellence Departments 2023–2027, MIUR (ARTICOLO 1, COMMI 314–337 LEGGE 232/2016), is gratefully acknowledged by authors of Roma Tre University.

## CRediT authorship contribution statement

**Stefania De Rosa:** Writing – review & editing, Writing – original draft, Methodology, Investigation, Data curation. **Elisabetta Colantoni:** Methodology, Investigation, Formal analysis, Data curation. **Paolo Branchini:** Resources, Project administration. **Domizia Orestano:** Resources, Project administration. **Antonio Passeri:** Resources, Project administration. **Gianlorenzo Bussetti:** Supervision, Investigation, Formal analysis, Data curation. **Lisa Centofante:** Methodology, Investigation. **Stefano Corradetti:** Writing – review & editing, Writing – original draft, Supervision, Methodology, Investigation, Data curation, Conceptualization. **Martina Marsotto:** Writing – review & editing, Investigation, Data curation. **Chiara Battocchio:** Writing – review & editing, Supervision, Investigation. **Cristina Riccucci:** Methodology, Investigation, Data curation. **Luca Tortora:** Writing – review & editing, Writing – original draft, Supervision, Resources, Project administration, Funding acquisition, Conceptualization.

## Declaration of competing interest

The authors declare that they have no known competing financial interests or personal relationships that could have appeared to influence the work reported in this paper.
