# Supplementary material for: Morphological and chemical changes in nuclear graphite target under vacuum and high-temperature conditions
Source: Heliyon. 2024 Jun 8;10(12):e32718. doi: 10.1016/j.heliyon.2024.e32718 (PMC11341341; doi:10.1016/j.heliyon.2024.e32718)
Supplement: Multimedia component 1 [file mmc1.docx]

**Supplementary Material**

**Morphological and chemical changes in nuclear graphite target under vacuum and high-temperature conditions**

Stefania De Rosa^a,b^, Elisabetta Colantoni^a,b,c^, Paolo Branchini^a,b^, Domizia Orestano^b,c^, Antonio Passeri^b^, Gianlorenzo Bussetti^d^, Lisa Centofante^e^, Stefano Corradetti^e^, Martina Marsotto^f^, Chiara Battocchio^f^, Cristina Riccucci^g^, and Luca Tortora^a,b,f*^

^a^ LASR3 Surface Analysis Laboratory Roma Tre, via della Vasca Navale 84, Rome, Italy;

^b^ INFN, Roma Tre, via della Vasca Navale 84, Rome, Italy;

^c^ Department of Mathematics and Physics, Roma Tre University, via della Vasca Navale 84, Rome, Italy;

^d^ Department of Physics, Politecnico di Milano, Piazza Leonardo da Vinci 32, I-20133 Milano, Italy;

^e^ Legnaro National Laboratories (INFN-LNL), Viale dell’Università 2, Legnaro, Padova, Italy;

^f^ Department of Sciences, Roma Tre University, Via della Vasca Navale 84, Rome, Italy.

^g^ CNR- ISMN, Institute for the Study of Nanostructured Materials, Via Salaria Km 29300, Montelibretti, Rome, Italy.

* Correspondence: [luca.tortora@uniroma3.it](mailto:luca.tortora@uniroma3.it)


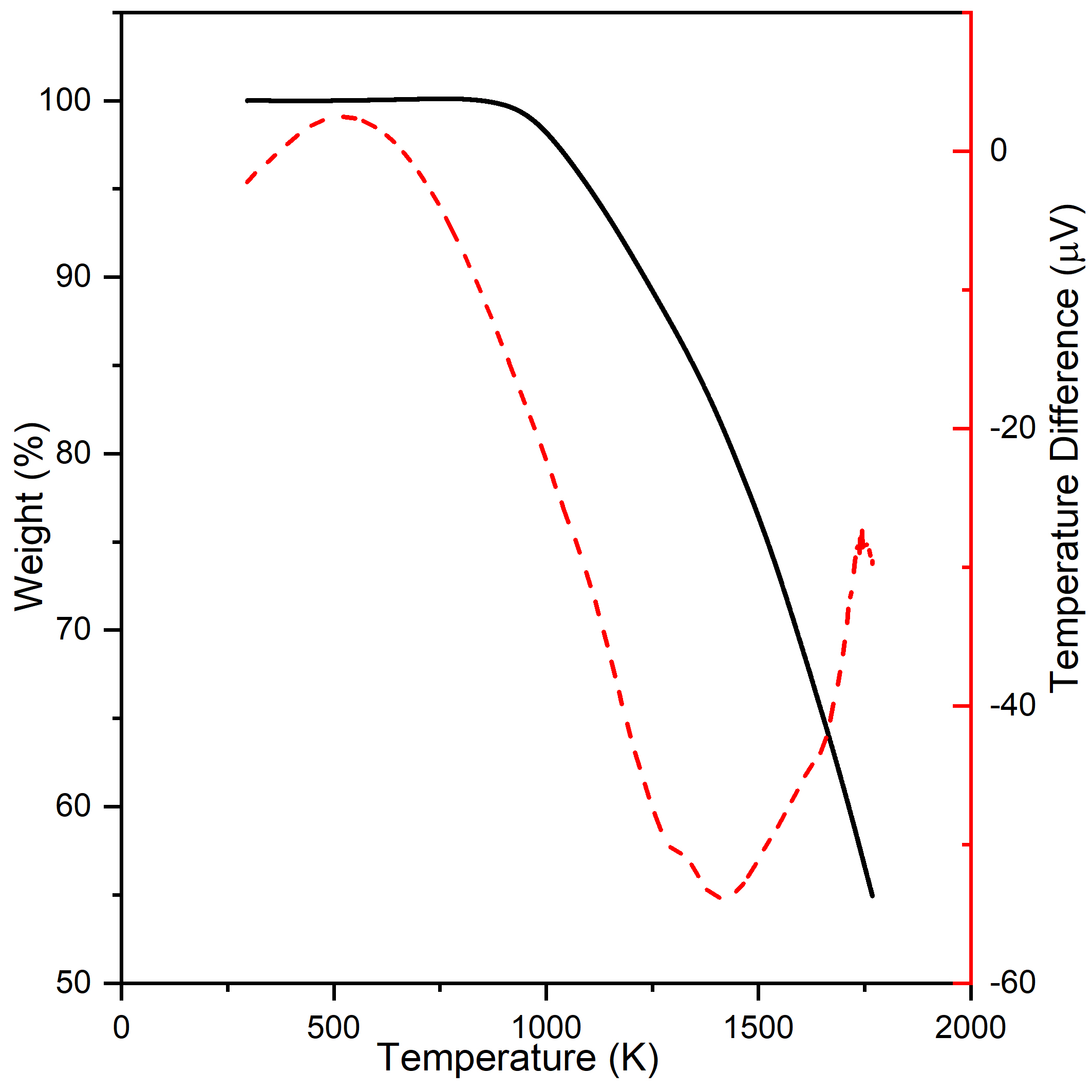


**Figure S1**. Weight loss and Temperature difference curves in N_2_ inert atmosphere of HPG59 graphite obtained with dynamic Thermo Gravimetry (TG) measurements.

**Table S1.** EDS analysis of pristine and heat-treated HPG59 graphite target. EDS spectra were collected from a 50 × 50 μm area within the images shown in Figure 2.

| Sample | C Wt % | O Wt % | Na Wt % | Al Wt % | Si Wt % | S Wt % | C/O ratio |
| --- | --- | --- | --- | --- | --- | --- | --- |
| Pristine graphite target | 49.87 | 31.48 | 12.15 | 3.26 | 0 | 3.24 | 1.58 |
| Treated graphite target (edge) | 85.04 | 8.62 | 1.07 | 1.65 | 2.66 | 0.97 | 9.87 |
| Treated graphite target (center) | 96.08 | 2.38 | 0.18 | 0.76 | 0 | 0.61 | 40.37 |


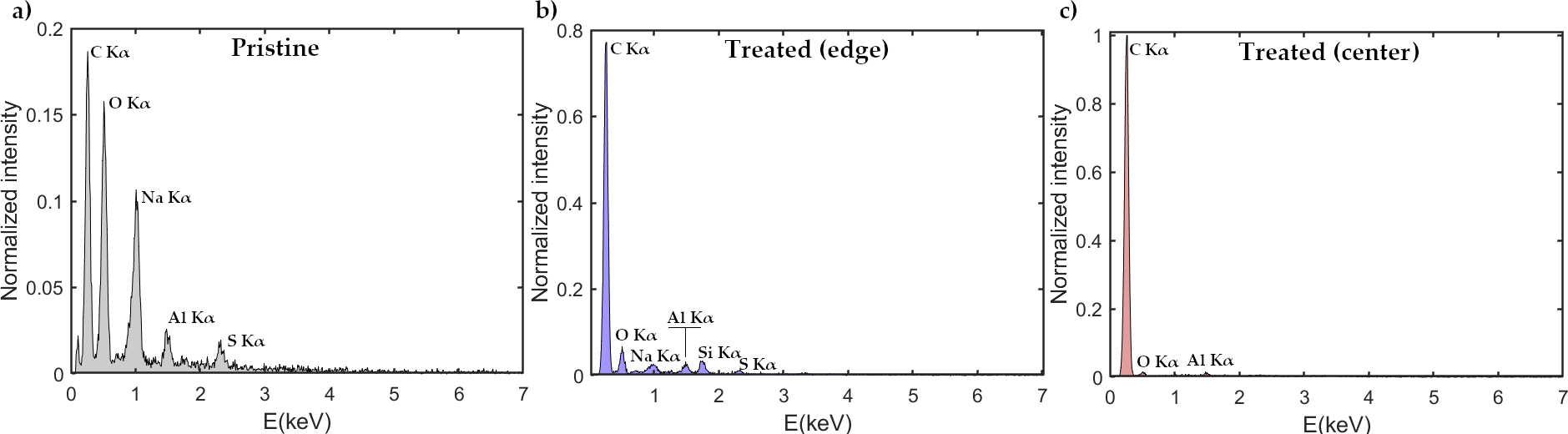


**Figure S2.** EDS spectra of (**a**) pristine graphite target, (**b**) heat-treated graphite target acquired at edge, and (**c**) heat-treated graphite target acquired at the center.

**
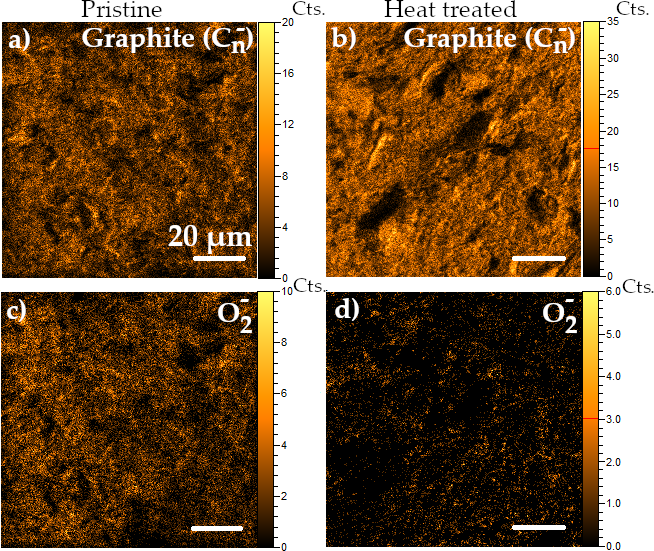
**

**Figure S3.** ToF-SIMS high lateral resolution ion maps (100 µm × 100 µm) showing the surface spatial distribution of (**a,b**) the sum of the C_n_^-^ (n=1-5) secondary ions of pristine and heat-treated graphite, and (**c,d**), O_2_^-^ secondary ions of pristine and heat-treated graphite.


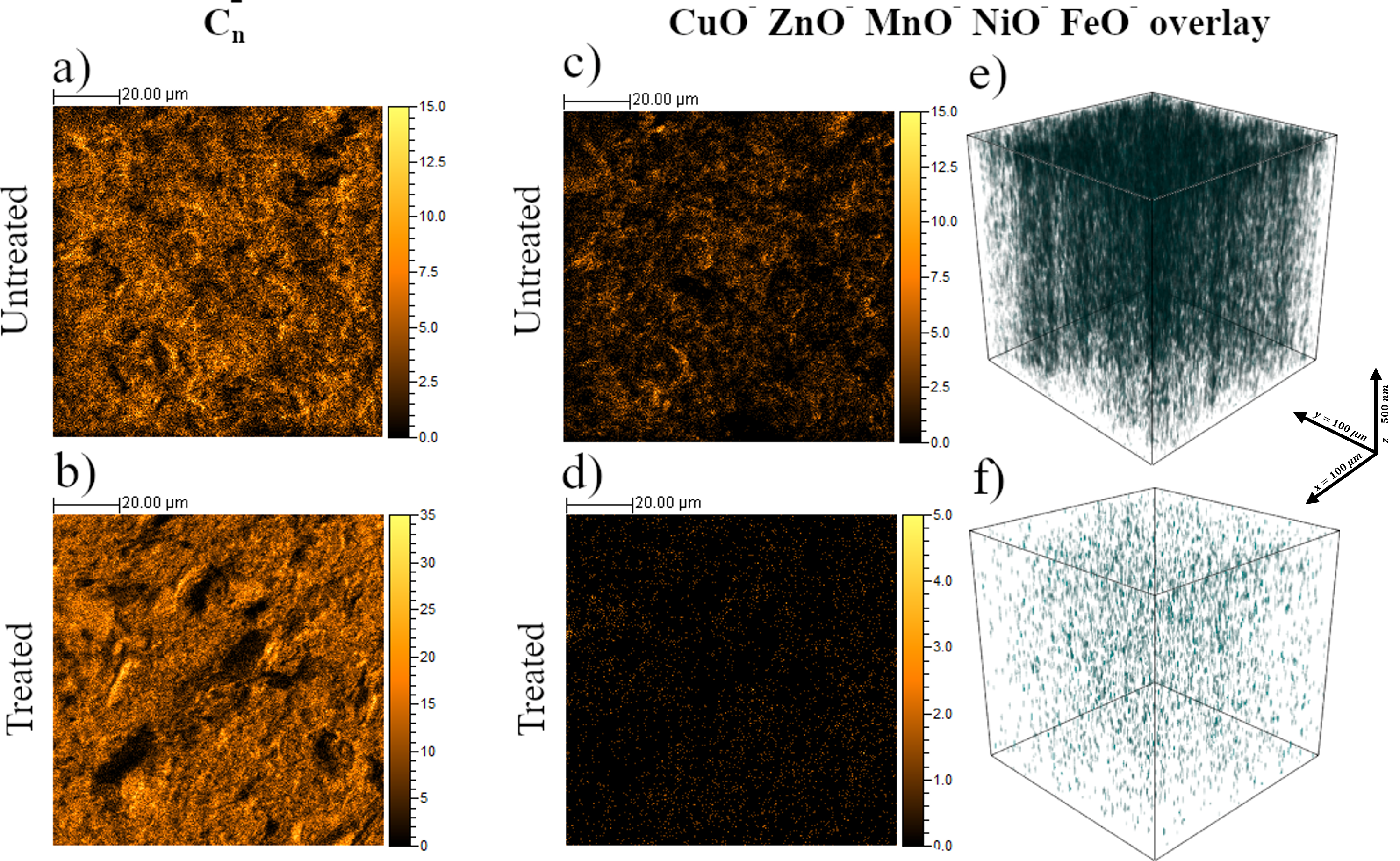


**Figure S4.** ToF-SIMS high lateral resolution ion maps (100 µm × 100 µm) showing the surface spatial distribution of the C_n_^-^ (n=1-5) secondary ions from untreated (a), and treated (b) samples. Surface (c,d) and in-depth (e,f) spatial distribution of CuO^-^, ZnO^-^, MnO^-^, NiO^-^, and FeO^-^ secondary ion intensity overlay of untreated (c, e), and treated (d,f) graphite target. Scale bar in (d) was modified to highlight the distribution of the signals. 3D data-cube reconstructions in (e,f) were obtained from ToF-SIMS depth profiling experiments.
